# Validation of Deep Capillary Plexus OCTA Metrics as Predictors of Diabetic Retinopathy Complications: A One-Year Longitudinal Study

**DOI:** 10.21203/rs.3.rs-9199781/v1

**Published:** 2026-06-28

**Authors:** Julia Greenwood, Shinji Kakihara, Anna Busza, Amani Fawzi

**Affiliations:** Northwestern University Feinberg School of Medicine; Northwestern University Feinberg School of Medicine; Northwestern University Feinberg School of Medicine; Northwestern University Feinberg School of Medicine

**Keywords:** diabetic retinopathy, optical coherence tomography angiography, deep capillary plexus, vessel density, retinal ischemia

## Abstract

Diabetic retinopathy (DR) can lead to vision-threatening complications, and tools that capture microvascular damage beyond standard clinical grading of disease severity may improve risk prediction. Optical coherence tomography angiography (OCTA) quantifies retinal perfusion, but its prognostic value in referable DR is not fully established. We aimed to validate baseline deep capillary plexus (DCP) OCTA metrics for predicting one-year complications in eyes with referable DR. In this prospective longitudinal study in 137 eyes of 96 participants, we assessed baseline predictors of DR complications, defined as best-corrected visual acuity (BCVA) loss (≥ 10 letters on the ETDRS chart), center-involving diabetic macular edema, anti-VEGF injections, pan-retinal photocoagulation, or vitreous hemorrhage. Baseline variables included BCVA, low-luminance visual acuity (LLVA), ocular parameters, demographic characteristics, systemic variables, and OCTA metrics (foveal avascular zone area, vessel density [VD] and geometric perfusion deficit in the superficial capillary plexuses [SCP] and [DCP]). Logistic regression prioritized variables with pathophysiologic relevance while minimizing risk of collinearity. Receiver operating characteristic (ROC) curves assessed whether DCP OCTA metrics improved discrimination beyond a clinical model with traditional risk factors. Over one year, 34 eyes (24.8%) experienced one or more complications. Multivariate analysis including DR severity, DCP VD, and LLVA identified higher baseline DR severity (OR, 5.77; 95% CI: 1.93 to 17.27; P = 0.002) and lower DCP VD (OR, 0.59; 95% CI: 0.36 to 0.95; P = 0.031) as significant predictors. Adding DCP VD to the clinical model significantly improved discrimination. These findings support DCP OCTA metrics as capillary-level biomarkers for risk stratification in referable DR and highlight the need for larger longitudinal studies to confirm clinical utility.

## Introduction

Diabetic retinopathy (DR) is a leading cause of vision loss and blindness worldwide, representing a significant global health burden.[1, 2] Disease progression significantly increases the risk of complications, such as diabetic macular edema (DME) and vitreous hemorrhage.[3] These complications not only lead to vision loss, but also impose considerable burden on patients’ quality of life, making early detection and timely intervention critical.[4]

Patients with referable DR, defined as moderate NPDR (characterized by multiple microaneurysms and hemorrhages) through PDR (presence of neovascularization),[5, 6] are a critical population that need referral to ophthalmologic care due to their heightened risk of disease progression and adverse outcomes.[7] The Early Treatment Diabetic Retinopathy Study (ETDRS) found that 46% of eyes with severe NPDR progress to PDR within a year,[8] while The Diabetic Retinopathy Study (DRS) found that without treatment, 25–35% of eyes with high-risk PDR experience severe vision loss (worse than 5/200) within two years.[9]

Clinical and systemic risk factors have traditionally been associated with DR development and progression. Epidemiological studies such as The Meta-Eye Analysis for Eye Disease (META-EYE) study group found that longer duration of diabetes, elevated HbA1c (poor glycemic control), and hypertension are major risk factors for the development of DR.[10] The Wisconsin Epidemiologic Study of Diabetic Retinopathy (WESDR) highlighted factors like poor glycemic control, smoking, and hypertension as significant contributors to DR progression.[11] Similarly, the Diabetes Control and Complications Trial (DCCT) in patients with type 1 diabetes and the United Kingdom Prospective Diabetes Study (UKDPS) in patients with type 2 diabetes demonstrated that improved glycemic control significantly reduces the risk of DR progression; however, glycemic exposure alone explained only 11% of the variation in DR progression, underscoring the complexity of predicting DR progression and complications.[12–15] These findings highlight the limitations of traditional risk factors, emphasizing the need for additional predictors to better understand and manage DR.

Recent advances in retinal imaging, particularly optical coherence tomography angiography (OCTA), offer a novel approach to assessing retinal microvascular changes.[16] Unlike fluorescein angiography (FA), OCTA allows for layer-specific visualization of the retinal capillary plexuses, enabling a more comprehensive evaluation of vascular abnormalities and ischemia without the risk of adverse reactions and need for intravenous dye administration.[17–20] OCTA metrics such as vessel density (VD), foveal avascular zone (FAZ) area, and geometric perfusion deficits (GPD) can quantify non-perfusion and thus serve as important markers for disease activity and progression. Numerous studies have demonstrated that OCTA nonperfusion metrics can differentiate DR severity, showing that VD declines, while FAZ and GPD increase with DR severity.[21–25]

Of the different OCTA metrics, a growing body of evidence suggests that deep capillary plexus (DCP) OCTA metrics are important predictors of DR severity progression and DR outcomes. Tsai and colleagues found that baseline FAZ area and VD within the DCP were associated with worse visual outcomes after one year.[26] Similarly, a 1-year prospective study by You and colleagues reported that extrafoveal avascular areas within the DCP were associated with the need for treatment, including anti-VEGF, steroids, and pan-retinal photocoagulation (PRP).[27] However, both studies included participants with a wide range of DR severity and did not adjust for disease severity, an important confounder when interpreting OCTA vascular changes. In a 2-year longitudinal study of 205 eyes, Sun and colleagues found that OCTA metrics in the DCP were associated with DR progression.[28] While this work adjusted for DR severity and other risk factors, all these prior studies did not include vitreous hemorrhage as a clinically relevant outcome.

Relevant to OCTA prediction, our group has previously demonstrated that in eyes with referable DR, both VD and GPD within the DCP could accurately predict 1-year complications, defined as center-involving edema, vitreous hemorrhage, or initiation of treatment with pan-retinal photocoagulation or anti-VEGF injections.[29] This work focused exclusively on treatment naïve referable NPDR eyes, incorporated detailed three-layer retinal segmentation (superficial, middle, and deep capillary plexuses), and adjusted for DR severity, allowing for more precise evaluation of OCTA metrics within a narrowly defined, high-risk population.

The goal of the present study is to validate and extend this prior work by analyzing a larger cohort (137 vs. 61 eyes), which spans the full spectrum of referable DR. Like our previous study, we adjust for DR severity to control for confounding but implement the simplified two-layer OCTA segmentation (SCP and DCP). To enhance generalizability, we also include clinically stable, previously treated PDR eyes and broaden the definition of DR complications by additionally including vision loss. Collectively, this design allows us to more rigorously test the predictive utility of DCP metrics across a representative cohort of patients with referable DR, validating their role as imaging biomarkers for risk stratification and management.

## Methods

### Study Design

This was a prospective longitudinal study conducted at the Department of Ophthalmology at Northwestern University in Chicago, Illinois from October 2021 to August 2024. The study was approved by the Institutional Review Board of Northwestern University and conducted in accordance with the regulations of the Health Insurance Portability and Accountability Act and the tenets of the Declaration of Helsinki. Written informed consent was obtained from all participants.

### Study Participants

Inclusion criteria were a diagnosis of type 1 or type 2 diabetes, the ability to give informed consent, and referable DR, defined as moderate nonproliferative DR (NPDR) to proliferative DR (PDR). Eyes were classified using the International Classification of Diabetic Retinopathy severity scale.[6] Moderate NPDR was defined as the presence of both microaneurysms and intraretinal hemorrhages. Severe NPDR was classified based on the 4-2-1 rule: >20 intraretinal hemorrhages in each of the four quadrants, venous beading in at least 2 quadrants, or prominent intraretinal microvascular abnormalities in at least one quadrant. PDR was defined by the presence of neovascularization or vitreous/preretinal hemorrhage. Specifically, participants were classified as PDR naïve (PDRn) if they have PDR but no prior laser treatment. Participants were classified as PDR quiescent (PDRq) if they have PDR that was previously treated with laser. Participants with PDRq were eligible if they had no active neovascularization for at least 6 months and had been stably treated with pan retinal photocoagulation. DR severity classifications in this study are based on the diagnosis at baseline visit.

Exclusion criteria were clinical evidence of diabetic macular edema (DME), defined as a central sub-field thickness (CST) ≥ 305 microns for women and ≥ 320 microns for men on OCT,[30] any history of intravitreal anti-VEGF or IVT steroid treatment in the last 6 months, and any ocular condition present that may affect the retinopathy status or alter visual acuity during the study. We excluded eyes with an axial length (AL) > 26 mm or < 22 mm (measured with IOLMaster), history of major ocular surgery (including cataract extraction, scleral buckle, any intraocular surgery) within the prior 3 months, or anticipated surgery within the next 6 months following enrollment. Eyes with an image quality score (Q-score) < 6 (1–10 scale) or obvious motion artifacts were excluded. Patients participating in a trial evaluating investigational medicinal products and those with an HbA1c > 10.0% were also excluded.

Patients were managed per standards of clinical care,[8] undergoing a comprehensive history, ophthalmic examination including dilated fundus examination, grading of DR and presence or absence of macular edema, and spectral domain-optical coherence tomography (SD-OCT, Heidelberg Spectralis) at baseline and one year. Early Treatment Diabetic Retinopathy Study (ETDRS) vision charts were used to measure best corrected visual acuity (BCVA) and low luminance visual acuity (LLVA) using standard operating procedures. Imaging and measurements were conducted at both baseline and one year.

### OCT Angiography Imaging

OCTA images were captured using the AngioVue OCTA system using the 3×3 mm (304 × 304 pixels) cube on the RTVue-XR Avanti OCT device (Optovue, Inc, version 2017.1.0.151) with split-spectrum amplitude-decorrelation angiography (SSADA) to extract angiographic flow data.[31] The machine operates at a wavelength of 840 nm with a scan speed of 70,000 A-scans per second, and a bandwidth of 50 nm. The tissue resolution is 5 μm axially with a 15 μm beam width. The OCTA performs two repeated B-scans from 304 sequentially uniformly spaced locations. Each B-scan consists of 304 A-scans, resulting in a total of 2 × 304 × 304 A-scans per acquisition. The entire scan acquisition is completed in approximately 3 seconds. The SSADA algorithm extracts motion contrast by quantifying decorrelation between the two successive B-scans. Motion correction is applied by the system by aligning the two orthogonal raster scans.

### OCTA Image Analysis

We utilized the default AngioVue software settings to segment the retinal vasculature into the superficial capillary plexus (SCP), deep capillary plexus (DCP), and full retinal slabs. The SCP slabs extend from the internal limiting membrane to 10 μm above the inner plexiform layer. The DCP slabs span from 10 μm above the inner plexiform layer to 10 μm below the outer plexiform layer. The full retinal slabs combine both layers, extending from the ILM to 10 μm below the outer plexiform layer. The scans were then exported into FIJI, an open-source distribution of ImageJ.[32] To align SCP angiograms, we used the Register virtual stack slices plugin with a rigid feature extraction model and elastic registration model. The best quality angiogram was chosen as a reference for registration. The transformation method was applied to the DCP and full retinal slabs using the Transform virtual stack slices plugin. Lastly, we enhanced image quality by averaging the aligned slabs in each stack using the average intensity projection in the Z Project plugin, a method known to reduce noise and improve image clarity.[33]

To assess OCTA nonperfusion parameters, the SCP, DCP and full retinal slabs were binarized using the global thresholding Huang2 plugin for capillaries and Max Entropy plugin for large vessels. We removed isolated noise pixels from the binarized slabs to prevent false vessel detection. Any vessels that became fragmented were reconnected with morphological operations, and the final image was rebinarized with mean thresholding. Capillaries were skeletonized because the transverse resolution of the OCTA system was insufficient to fully resolve their structure. Vessel density (VD) was calculated from binarized averaged images and defined as the percentage of white pixel area divided by the total 3×3 mm image area. The FAZ was manually traced from the full retinal slab using the Polygon selection tool in ImageJ.

**Figure 1:**
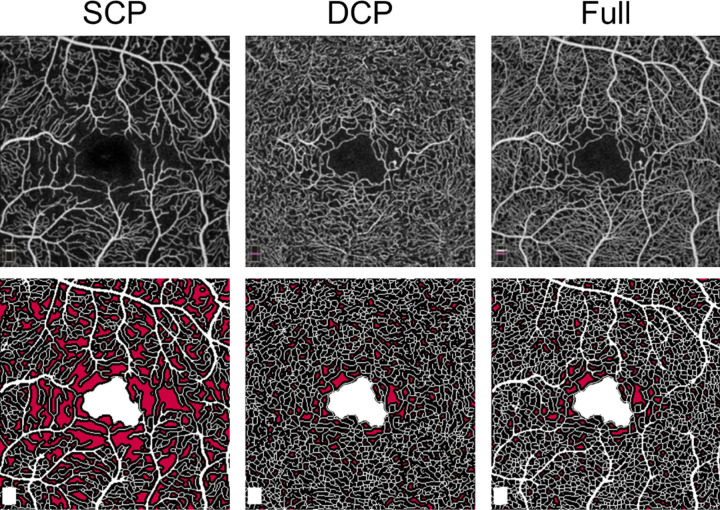
Representative determination of geometric perfusion deficit (GPD) and foveal avascular zone (FAZ) from combined 3×3 mm en face OCTA scans using Fiji. Top row: Aligned slabs combining five OCTA images from the superficial capillary plexus (SCP, left), deep capillary plexus (DCP, middle), and full retinal slabs (right). Bottom row: GPD (red areas) represents the percentage of the area >30 μm from the nearest blood vessel, excluding the FAZ (central white areas). For DCP measurements, areas beneath large vessels were excluded from GPD and total area calculations to minimize the effect of projection and shadowing artifacts.

We used a semiautomated FIJI macro to measure geometric perfusion deficit (GPD) in averaged scans of the SCP and DCP (Fig. 1). GPD was defined as the percentage of area > 30 μm away from the nearest blood vessel,[34] a threshold based on prior studies of oxygen diffusion in animal models and parafoveal intercapillary distances.[35–37] A large vessel mask was applied to the skeletonized SCP and full capillary angiogram before the GPD measurement to prevent misclassification of large vessels as GPD areas. Since overlaying large vessels onto the DCP may artificially erase true GPD areas due to projection and shadowing artifacts, GPD was measured using the skeletonized DCP angiogram. Areas beneath large vessels were then subtracted from both GPD and total area calculations to ensure measurement accuracy. The FAZ area and watermark were omitted from all GPD calculations.

### DR Complications and Baseline Variables

DR complications were defined as one or more of the following: (1) BCVA loss (≧ 10 letters according to the Early Treatment Diabetic Retinopathy Study [ETDRS] protocol), (2) center-involving diabetic macular edema (central subfield thickness [CMT] ≥ 320 μm for males, ≥ 305 μm for females), (3) anti-VEGF injections, (4) pan-retinal photocoagulation, or (5) vitreous hemorrhage.

Predictors analyzed include baseline visual function (best-corrected visual acuity [BCVA], low-luminance visual acuity [LLVA]), ocular parameters (axial length [AL], lens status), demographic characteristics (age, gender, ethnicity, diabetes type), systemic variables (diabetes duration, HbA1c, hypertension [HTN], dyslipidemia, smoking, ischemic heart disease, renal disease, cerebrovascular disease), DR severity, and OCT angiography metrics (foveal avascular zone [FAZ] area, vessel density [VD] in the superficial and deep capillary plexuses [SCP, DCP], and geometric perfusion deficit [GPD] in the SCP and DCP). Systemic variables were obtained by patient interview and chart review.

### Statistical Analysis

Statistical analysis was performed using R (R version 4.2.2; R Project for Statistical Computing). Intraclass correlation coefficients were calculated to determine inter-rater reliability between different graders (J.I.G. and S.K.) for FAZ area measurements in 20 eyes. Shapiro-Wilk tests were performed to determine whether continuous data were normally distributed. Demographic and clinical data were compared with independent-sample t tests for normally distributed data, Mann-Whitney U tests for nonparametric data, and Fisher exact tests for categorical data. Descriptive statistics include mean, standard deviation (SD), and percentages.

To identify predictors of DR complications after one year, we performed mixed effects univariate logistic regression analysis to account for clustering of eyes within participants. Mixed effects multivariate logistic regression analysis was then performed with select variables found to be significant in the univariate logistic regression, prioritizing variables with pathophysiologic relevance while minimizing risk of co-linearity. For DCP imaging parameters, receiver operating characteristic (ROC) curves were constructed to determine the area under the curve (AUC). Optimal cutoffs on the ROC curves were defined as thresholds that maximized Youden’s index (sensitivity + specificity – 1). We used DeLong’s nonparametric test for pairwise comparisons of DCP AUCs.[38] We also constructed a baseline clinical model including traditional DR risk factors including diabetes duration, HbA1c, and hypertension as covariates to evaluate the added predictive value of VD-DCP. P-values of 0.05 were considered statistically significant for all statistical tests.

## Results

Within the study period, 129 patients (180 eyes) qualified based on the inclusion and exclusion criteria. Of these participants, 96 patients (137 eyes) returned for 1-year follow-up. All demographic, clinical, and imaging characteristics were similar between returning patients and patients lost to follow-up except for ethnicity (p = 0.046) and baseline best-corrected visual acuity (BCVA, p = 0.024) (Table 1). The intergrader for FAZ area measurements was excellent (intraclass correlation coefficient = 0.998; 95% confidence interval [CI], 0.995–1.000; P < 0.001), validating the method’s reliability.

At 1 year, 34 eyes (24.8%) experienced complications. Five eyes developed BCVA loss ≥ 10 letters and 18 eyes developed center-involving DME. Four eyes received anti-VEGF injections, 13 eyes received PRP, and 10 eyes experienced vitreous hemorrhage. Several eyes experienced more than one clinically significant event, with a total of 50 clinically significant events from the 34 eyes. For instance, 1 eye experienced BCVA loss, DME, and received anti-VEGF; 3 eyes developed DME, vitreous hemorrhage, and received PRP; and 4 eyes developed both vitreous hemorrhage and received PRP. Complications were disproportionately observed in eyes with more advanced DR severity. Of the 34 eyes with complications, 1 eye had moderate NPDR at baseline, 5 eyes had severe NPDR, 9 had naïve PDR, and 19 had quiescent PDR (Table 2).

Demographic characteristics did not differ between patients who developed complications and those who did not. Clinical characteristics such as baseline BCVA (P = 0.009), LLVA (P = 0.028), and DR severity (P < 0.001) showed notable differences. Mann-Whitney U and independent t-tests revealed significant differences on OCTA imaging metrics, including FAZ area (P = 0.021), GPD in the SCP (P = 0.011) and DCP (P = 0.014), VD in the SCP (P < 0.001) and DCP (P < 0.001), and Q-score (P = 0.022) (Table 3).

Univariate logistic regression with mixed effects was used to assess the association between each baseline variable and the likelihood of developing complications. DR severity (odds ratio [OR], 7.06 odds increase for PDR; 95% CI, 2.69–18.54; P < 0.001), BCVA (OR, 0.46 per 10-letter increase; 95% CI, 0.27–0.80; P = 0.006), LLVA (OR, 0.52 per 10-letter increase; 95% CI, 0.32–0.87; P = 0.006), GPD-DCP (OR, 1.75 per 5% increase; 95% CI, 1.04–2.94; P = 0.02), and VD-DCP (OR, 0.46 per 5% increase; 95% CI, 0.30–0.69; P < 0.001) were statistically significant in the univariate analysis (Table 4). To allow for direct comparison of effect sizes, baseline variables were also standardized using z-score transformation such that the ORs reflect the change in odds per one standard deviation (SD) in each predictor (**Supplementary Table 1**). The standardized analysis yielded results consistent with the original analysis, showing similar associations with DR complications.

Multivariate logistic regression with mixed effects was then performed to identify independent predictors of DR complications, while adjusting for potential confounders. Due to the limited number of complications at one year (n = 34), we limited the multivariate model to three variables to maintain an appropriate event per variable ratio and avoid overfitting. DR severity was included because it had the strongest association with complications in the univariate analysis (OR, 7.06; 95% CI, 2.85–20.23; P < 0.001) and has long been established as a key predictor of progression and complications.[21–25] Only one OCTA imaging metric was included in the multivariate model to avoid co-linearity. VD was selected since it performed better than GPD in the univariate analysis. VD-DCP was chosen over VD-SCP given their comparable univariate performance and prior studies suggesting that DR preferentially affects the deep capillary plexus.[24, 25, 39, 40] Of the remaining significant variables from the univariate analysis, LLVA was prioritized since recent studies have shown that LLVA is associated with retinal non-perfusion. [41, 42] DR severity (OR, 5.84 odds increase for PDR; 95% CI, 2.00–17.09; P = 0.001) and VD-DCP (OR, 0.59 per 5% increase; 95% CI, 0.36–0.96; P = 0.037) remained significant, while LLVA (OR, 0.88 per 10-letter increase; 95% CI, 0.56–1.40; P = 0.604) did not (Table 5).

ROC curve analysis was performed to further explore how well DCP metrics predict DR complications compared to traditional DR risk factors. Univariate ROC analysis showed VD and GPD in the DCP as predicting clinically significant outcomes (P < 0.01). VD achieved a slightly higher AUC compared to GPD (AUC = 0.714 vs 0.645, respectively), which was not statistically significant (Delong’s method, P = 0.215). At cutoffs selected by maximizing Youden’s index, sensitivity was 67.6% (95% CI, 49.5–82.6%) and specificity 73.8% (95% CI 64.2–82.0%) for VD-DCP ≤ 44.9%, and sensitivity was 61.8 (95% CI, 43.6–77.8%) and specificity 62.1% (95% CI, 52.0–71.5%) for GPD-DCP ≥ 5.89%.

A baseline clinical ROC model of diabetes duration, HbA1c, and hypertension, well-established risk factors for DR development and progression,[10] yielded an AUC of 0.551. Adding VD-DCP to the model significantly improved the AUC to 0.715 (ΔAUC = 0.164, P = 0.017).

## Discussion

In this study, we evaluated how baseline patient characteristics correlated with 1-year outcomes in eyes with referable DR, with the aim to validate DCP metrics as predictive biomarkers. In a multivariate model incorporating DR severity, VD-DCP, and LLVA, both DR severity and VD-DCP remained significant predictors of complications (Table 5). ROC analysis demonstrated that both VD and GPD in the DCP significantly discriminated eyes that developed complications, with no difference in their predictive performance. Addition of VD-DCP to a base clinical model with diabetes duration, HbA1c, and hypertension yielded a significant improvement in predictive ability, supporting that DCP OCTA metrics capture microvascular changes beyond traditional DR risk factors. Our findings that lower VD-DCP and higher GPD-DCP distinguish eyes that develop complications are consistent with previous studies and extends these works by adjusting for DR severity.[26–28] This study also aligns with our group’s previous work which identified DCP metrics as significant predictors of DR complications, and expands it to a broader definition of complications that incorporates BCVA loss.[29]

Interestingly, the predictive performance of VD-DCP and GPD-DCP in our study yielded lower AUCs than our previous report. Differences in discriminative ability may be due to differences in methodology and the study cohorts. First, while this work used the more conventional two-layer segmentation, Ong et al. segmented the vasculature into three layers (SCP, MCP, DCP). While this study’s segmentation grouped the MCP with the DCP, which is preferable to including it with the SCP[43], this may have contributed to the variation in reported AUCs.[29] Second, Ong et al. analyzed single OCTA scans per eye, while our study used an image averaging approach with multiple scans to generate en face images, a method shown to improve image quality and signal-to-noise ratio.[33] Additionally, VD was assessed across the full 3×3 mm image in this study, while previously VD was calculated within an annular area between the concentric 1 mm and 3 mm rings centered on the fovea. Differences in patient populations, particularly our inclusion of previously treated PDR eyes, introduced greater clinical heterogeneity and may have also contributed to differences in our findings. Interestingly, our study observed a slightly lower complication rate (24.8%) compared to our previous study (29.5%) despite including eyes with PDR and employing a broader definition of complications which include BCVA loss. Notably, only six of 34 complications in our cohort occurred in NPDR eyes, and only five eyes experienced BCVA loss, suggesting that most complications arose from PDR eyes and that functional visual decline occurred in a minority of cases. Despite these differences, our findings agree that DCP OCTA metrics can be used as biomarkers for complications of DR, validating the findings in our previous work.

Our finding that VD-DCP remained a significant predictor of complications in the multivariate analysis aligns with the current understanding of DR pathophysiology.[44–51] Chronic hyperglycemia in diabetes leads to microvascular dysfunction, including endothelial cell damage, thickening of the basement membrane, and pericyte loss, all of which compromise capillary structure and perfusion.[44] Physiologic features of the DCP make it particularly vulnerable to decreased VD and increased GPD in DR. The DCP is situated at the interface of the inner nuclear and outer plexiform layers of the retina, which are highly metabolically active and thus are particularly susceptible to ischemic injury. This vulnerability is in part due to the DCP’s relative distance from the retinal arterioles and its association with the venous side of circulation, where perfusion pressure is lower.[45, 46] Histological analysis by our group has shown that pericytes, key regulators of retinal blood flow and vascular stability, are less densely distributed in the DCP (20.1 per 1000 μm of retinal capillary) compared to the SCP (22.9 per 1000 μm) in healthy eyes. The lower pericyte density in the DCP may underlie its increased vulnerability to perfusion deficits and ischemic injury in DR.[47] The DCP is also in close proximity to the photoreceptor layer, which has been found to be a prominent source of oxidative stress.[48] Additionally, the outer retina lies in a watershed zone between the retinal and choroidal circulations, making it more prone to hypoxia.[49] Hypoxic stress in this region stimulates upregulation of VEGF and other hypoxia-inducible factors, promoting further vascular instability.[50, 51] Collectively, these mechanisms highlight the potential mechanistic underpinnings of VD-DCP correlation with risk of DR complications.

The inclusion of eyes with NPDR and PDR in this study may be viewed as a limitation due to the heterogeneity in disease severity. Including eyes with PDR enables a more comprehensive evaluation of predictors across the full range of referable DR, enhancing the generalizability of our findings to diverse clinical populations. While stratifying risk within NPDR eyes is crucial for strategies aimed at preventing complications, identifying high-risk cases among PDR eyes is important to focus on those who require more intensive surveillance because of their higher risk to vision-threatening complications such as vitreous hemorrhage. Moreover, by including DR severity as a covariate in our multivariate analysis, we reduced potential confounding effects, allowing us to validate the value of OCTA metrics in stratifying risk of DR complications.

While prior studies largely focused on individual outcomes such as DR progression or DME development, we used a pooled definition of DR complications. This composite endpoint improved the statistical power by increasing the complication rate, and capturing the diverse range of clinically significant outcomes in referable DR. However, we acknowledge that these components differ in clinical significance and rate, and future studies in larger cohorts should explore their individual predictive associations to further refine risk stratification. Other limitations include clinical variation in management strategies, as patients were managed by different physicians. Future studies with larger cohorts and extended follow-up periods are warranted to expand and build upon our work.

Our study successfully validated DCP OCTA nonperfusion metrics as significantly associated with the development of DR complications in patients with referable DR and provided significantly enhanced discriminative ability in models with traditional clinical risk factors. This underscores the importance of capillary level metrics in DR progression and the need for further investigations with larger samples.

## Supplementary Files

This is a list of supplementary files associated with this preprint. Click to download.

- scientificreportssupplementary.docx

## Figures and Tables

**Table 1:** Demographic, Clinical, and Imaging Characteristics of Returning and Lost to Follow-up Patients

Subject Characteristics	Returning (n = 96; 137 eyes)	Lost to follow-up (n = 33; 43 eyes)	P-value
Age (years), mean ± SD	61.1 ±11.9	57.0 ± 12.0	0.074
Gender, male/female (% female)	47/49 (51.0)	17/16 (48.5)	0.842
Ethnicity			0.046*
Hispanic/Latino, n (%)	36 (37.5)	8 (24.2)	
Not Hispanic/Latino, n (%)	59 (61.5)	22 (66.7)	
Prefer not to answer, n (%)	1 (1.0)	3 (9.1)	
DM type, type 1/type 2 (% type 1)	21/75 (21.9)	7/26 (21.2)	1.00
DM duration (years), mean ± SD	25.9 ± 13.1	24.7 ± 12.8	0.992
HbA1c (%), mean ± SD	7.36 ± 1.31	7.43 ± 1.24	0.957
DR severity NPDR/PDR (% PDR)	68/69 (50.3)	21/22 (51.1)	1.00
CMT (um), mean ± SD	272 ± 55.5	260 ± 48.7	0.151
Hypertension, n (%)	72 (75.0)	25 (75.8)	1.00
Ischemic heart disease, n (%)	25 (26.0)	9 (27.3)	1.00
Renal disease, n (%)	22 (22.9)	12 (36.4)	0.149
Cerebrovascular disease, n (%)	8 (8.3)	1 (3.0)	0.446
Dyslipidemia, n (%)	60 (62.5)	20 (60.6)	0.839
Smoking history (%)			0.298
None	61 (63.5)	17 (51.5)	
Past	30 (31.3)	12 (36.4)	
Current	5 (5.2)	4 (12.1)	
Eye laterality, OD/OS (% OD)	67/70 (48.9)	24/19 (55.6)	0.486
Baseline BCVA (ETDRS), mean ± SD	79.1 ±7.00	81.3 ± 8.16	0.024*
Baseline LLVA (ETDRS), mean ± SD	70.6 ± 7.81	72.6 ± 8.54	0.101
Baseline LLVAD (ETDRS), mean ± SD	8.5 ± 3.72	8.7 ± 4.04	0.722
Axial length (mm), mean ± SD	23.4 ± 0.92	23.6 ± 0.91	0.239
FAZ area (mm^2^), mean ± SD	0.40 ± 0.23	0.44 ± 0.23	0.224
GPD (%), mean ± SD			
SCP	14.0 ± 5.00	14.4 ± 7.57	0.953
DCP	6.69 ± 3.39	6.60 ± 4.25	0.435
Vessel density (%), mean ± SD			
SCP	39.4 ± 5.32	0.40 ± 0.07	0.781
DCP	44.3 ± 4.89	0.45 ± 0.05	0.075
Q-score, mean ± SD	7.53 ± 0.96	7.72 ± 0.99	0.214

Abbreviations: DM = diabetes mellitus; DR = diabetic retinopathy; NPDR = nonproliferative diabetic retinopathy; PDR = proliferative diabetic retinopathy; CMT = central macular thickness; BCVA = best-corrected visual acuity; LLVA = low luminance best-corrected visual acuity; LLVAD = low luminance best-corrected visual acuity difference; OD = oculus dexter; OS = oculus sinister; FAZ = foveal avascular zone; GPD = geometric perfusion deficit; VD = vascular density; SCP = superficial capillary plexus; DCP = deep capillary plexus; SD = standard deviation.

* P-value indicates statistical significance of the test (P ≤ 0.05).

**Table 2:** Total Number of Eyes with Complications by DR Severity

DR Severity	Total Complications	BCVA loss	CME	Anti-VEGF	PRP	vitreous hemorrhage	Total events
moderate NPDR	1	1	1	1	0	0	3
severe NPDR	5	0	5	1	0	0	6
naïve PDR	9	2	3	0	7	2	14
quiescent PDR	19	2	9	2	6	8	27
Total	34	5	18	4	13	10	50

Abbreviations: DR = diabetic retinopathy; BCVA = best-corrected visual acuity; CME = central macular edema; VEGF = vascular endothelial growth factor; PRP = panretinal photocoagulation; NPDR = nonproliferative diabetic retinopathy; PDR = proliferative diabetic retinopathy.

**Table 3:** Demographic, Clinical, and Imaging Characteristics of Study Patients

Subject Characteristics	No Complications (n = 64; 103 eyes)	Complications (n = 32; 34 eyes)	P-value
Age (years), mean ± SD	61.2 ± 11.1	58.6 ± 13.1	0.303
Gender, male/female (% female)	34/30 (46.9)	13/19 (40.6)	0.261
Ethnicity			0.150
Hispanic/Latino, n (%)	21 (32.8)	15 (46.9)	
Not Hispanic/Latino, n (%)	42 (65.6)	17 (53.1)	
Prefer not to answer, n (%)	1 (1.6)	0	
DM type, type 1/type 2 (% type 1)	16/48 (25)	5/27 (15.6)	0.592
DM duration (years), mean ± SD	24.7 ± 12.9	24.5 ± 13.0	0.641
HbA1c (%), mean ± SD	7.39 ± 1.13	7.62 ± 1.20	0.629
DR severity NPDR/PDR (% PDR)	62/41 (40.0)	6/28 (82.4)	< 0.001*
CMT (um), mean ± SD	256.4 ± 51.8	271.3 ± 36.0	0.091
Hypertension, n (%)	46 (71.9)	26 (81.3)	0.610
Ischemic heart disease, n (%)	13 (20.3)	12 (37.5)	0.288
Renal disease, n (%)	13 (20.3)	9 (28.1)	0.288
Cerebrovascular disease, n (%)	6 (9.4)	2 (6.3)	1.00
Dyslipidemia, n (%)	40 (62.5)	20 (62.5)	1.00
Smoking history (%)			0.536
None	40 (62.5)	21 (65.6)	
Past	20 (31.3)	10 (31.3)	
Current	1 (1.6)	1 (3.1)	
Eye laterality, OD/OS (% OD)	50/53 (48.5)	17/17 (50)	1.000
Baseline BCVA (ETDRS), mean ± SD	82.6 ± 7.1	77.3 ± 10.0	0.009*
Baseline LLVA (ETDRS), mean ± SD	73.7 ± 7.7	69.0 ± 10.0	0.028*
Baseline LLVAD (ETDRS), mean ± SD	8.9 ± 4.1	8.3 ± 3.7	0.575
Axial length (mm), mean ± SD	23.7 ± 0.9	23.3 ± 0.9	0.303
FAZ area (mm^2^), mean ± SD	0.411 ± 0.218	0.531 ± 0.286	0.021*
GPD (%), mean ± SD			
SCP	0.135 ± 0.073	0.171 ± 0.078	0.011*
DCP	0.610 ± 0.039	0.081 ± 0.050	0.014*
VD (%), mean ± SD			
SCP	0.411 ± 0.067	0.355 ± 0.059	< 0.001*
DCP	0.463 ± 0.042	0.422 ± 0.063	< 0.001*
Complication		34	
Visual acuity loss, n (%)	-	5 (14.7)	
CME, n (%)	-	18 (52.9)	
anti-VEGF injections, n (%)	-	4 (11.8)	
Pan-retinal photocoagulation, n (%)	-	13 (38.2)	
Vitreous hemorrhage, n (%)	-	10 (29.4)	
Q-score, mean ± SD	7.84 ± 0.89	7.38 ± 1.18	0.022*

Abbreviations: DM = diabetes mellitus; DR = diabetic retinopathy; NPDR = nonproliferative diabetic retinopathy; PDR = proliferative diabetic retinopathy; CMT = central macular thickness; BCVA = best-corrected visual acuity; LLVA = low luminance best-corrected visual acuity; LLVAD = low luminance best-corrected visual acuity difference; FAZ = foveal avascular zone; GPD = geometric perfusion deficit; VD = vascular density; SCP = superficial capillary plexus; DCP = deep capillary plexus; CME = central macular edema; OD = oculus dexter; OS = oculus sinister; SD = standard deviation.

* P-value indicates statistical significance (P ≤ 0.05).

**Table 4:** Univariate Logistic Regression of Baseline Predictors

Subject Characteristics	OR (95% CI)	P-value
Age (years)	0.98 (0.95–1.01)	0.282
Gender	0.56 (0.23–1.36)	0.200
Ethnicity	0.48 (0.19–1.21)	0.120
DM type	1.90 (0.61–5.91)	0.264
DM duration (years)	0.99 (0.96–1.03)	0.667
HbA1c (%)	1.12 (0.76–1.64)	0.581
DR severity NPDR/PDR	7.06 (2.69–18.54)	<0.001*
CMT (um)	1.10 (0.96–1.27)	0.161
Hypertension	1.66 (0.58–4.74)	0.340
Ischemic heart disease	1.76 (0.65–4.78)	0.266
Renal disease	1.92 (0.63–5.80)	0.250
Cerebrovascular disease	0.50 (0.09–2.73)	0.424
Dyslipidemia	0.92 (0.38–2.22)	0.858
Smoking history	0.73 (0.34–1.59)	0.429
BCVA (ETDRS)	0.46 (0.27–0.80)	0.006*
LLVA (ETDRS)	0.52 (0.32–0.87)	0.012*
LLVAD (ETDRS)	0.66 (0.21–2.02)	0.469
Axial length (mm)	0.51 (0.29–0.91)	0.022*
FAZ area (mm^2^)	1.22 (1.03–1.47)	0.025*
GPD (%)		
SCP	1.40 (1.02–1.92)	0.039*
DCP	1.75 (1.04–2.94)	0.036*
Vessel density (%)		
SCP	0.50 (0.33–0.77)	0.002*
DCP	0.46 (0.30–0.69)	<0.001*

Abbreviations: DM = diabetes mellitus; DR = diabetic retinopathy; NPDR = nonproliferative diabetic retinopathy; PDR = proliferative diabetic retinopathy; CMT = central macular thickness; BCVA = best-corrected visual acuity; LLVA = low luminance best-corrected visual acuity; LLVAD = low luminance best-corrected visual acuity difference; OD = oculus dexter; OS = oculus sinister; FAZ = foveal avascular zone; GPD = geometric perfusion deficit; VD = vascular density; SCP = superficial capillary plexus; DCP = deep capillary plexus; SD = standard deviation.

* P-value indicates statistical significance (P ≤ 0.05).

**Table 5:** Multivariate Logistic Regression of Mixed Effects

Subject Characteristics	OR (95% CI)	P-value
DR Severity (NPDR/PDR)	5.84 (2.00–17.09)	0.001*
VD-DCP (%)	0.59 (0.36–0.96)	0.037*
LLVA (ETDRS)	0.88 (0.56–1.40)	0.604

Abbreviations: DR = diabetic retinopathy; NPDR = nonproliferative diabetic retinopathy; PDR = proliferative diabetic retinopathy; VD = vessel density; DCP = deep capillary plexus; BCVA = best-corrected visual acuity.

* P-value indicates statistical significance (P ≤ 0.05).

## Data Availability

The original contributions presented in this study are included in the article/supplementary material. Further inquiries can be directed to the corresponding author.
